# Neuronal hemoglobin affects dopaminergic cells' response to stress

**DOI:** 10.1038/cddis.2016.458

**Published:** 2017-01-05

**Authors:** Marta Codrich, Maria Bertuzzi, Roberta Russo, Margherita Francescatto, Stefano Espinoza, Lorena Zentilin, Mauro Giacca, Daniela Cesselli, Antonio Paolo Beltrami, Paolo Ascenzi, Silvia Zucchelli, Francesca Persichetti, Giampiero Leanza, Stefano Gustincich

**Affiliations:** 1 Area of Neuroscience, Scuola Internazionale Superiore di Studi Avanzati (SISSA), via Bonomea 265, Trieste 34136, Italy; 2 Department of Health Sciences, University of Eastern Piedmont ‘A. Avogadro', via Solaroli 17, 28100 Novara, Italy; 3 Department of Neuroscience and Brain Technologies, Istituto Italiano di Tecnologia (IIT), via Morego 30, Genova 16163, Italy; 4 ICGEB, Padriciano 99, 34149 Trieste, Italy; 5 Department of Medical and Biological Sciences, University of Udine, Piazzale Kolbe 4, Udine, 33100, Italy; 6 Department of Sciences, University of Roma Tre, viale G. Marconi 446, Roma 00146, Italy; 7 Department of Life Sciences, University of Trieste, via Fleming 22, Trieste 34127, Italy

## Abstract

Hemoglobin (Hb) is the major protein in erythrocytes and carries oxygen (O_2_) throughout the body. Recently, Hb has been found synthesized in atypical sites, including the brain. Hb is highly expressed in A9 dopaminergic (DA) neurons of the substantia nigra (SN), whose selective degeneration leads to Parkinson's disease (PD). Here we show that Hb confers DA cells' susceptibility to 1-methyl-4-phenylpyridinium (MPP^+^) and rotenone, neurochemical cellular models of PD. The toxic property of Hb does not depend on O_2_ binding and is associated with insoluble aggregate formation in the nucleolus. Neurochemical stress induces epigenetic modifications, nucleolar alterations and autophagy inhibition that depend on Hb expression. When adeno-associated viruses carrying *α*- and *β*-chains of Hb are stereotaxically injected into mouse SN, Hb forms aggregates and causes motor learning impairment. These results position Hb as a potential player in DA cells' homeostasis and dysfunction in PD.

Parkinson's disease (PD) is a chronic progressive neurodegenerative disorder clinically defined in terms of motor symptoms. The most evident pathological hallmarks are the selective degeneration of A9 dopaminergic (DA) neurons of substantia nigra pars compacta (SNpc) and the presence of intracellular aggregates called Lewy bodies. The consequent loss of DA synapses in the striatum is the primary origin of the inability to control movements.^1^ The causes of the selective degeneration of A9 neurons remain unknown.^2^

The identification of genes involved in early-onset PD has proved the crucial role of mitochondria,^1^ of the ubiquitin-proteasome system^1^ and autophagy.^3^

Epidemiological data showed that rural areas with heavy use of pesticides present a higher incidence of PD.^4^ The insecticide rotenone and the neurotoxin precursor 1-methyl-4-phenyl-1,2,3,6-tetrahydropyridine (MPTP) have been extensively used as PD-mimicking models to study the molecular mechanisms of DA cells' degeneration.^5, 6^ Both of them provoke depletion of intracellular ATP intervening on the homeostasis of mitochondrial activity and autophagy.^7, 8, 9^

In this context, the structure and function of the nucleolus have been found altered in *post mortem* PD brains. A correct ribosome biogenesis is essential to the survival of DA neurons *in vivo*^10^ while nucleolar damage is present in mouse DA cells upon MPTP intoxication.^11^

In the quests for the molecular basis of DA cells' dysfunction in PD, interesting cues have been obtained by comparing the molecular constituents of A9 DA cells of the SN with those of the adjacent A10 DA neurons of the ventral tegmental area. As these cells do not degenerate in PD, differential gene expression analysis has been associated with A9 susceptibility in disease. Examination of gene categories has highlighted that genes encoding energy-related metabolism, electron transport and mitochondria proteins appear to be more expressed in A9 rather than A10 neurons.^2^

We and others have previously identified the transcripts of *α*- and *β*-chains of hemoglobin (Hb) in A9 DA neurons.^12, 13^ Hb immunoreactivity decorates the large majority of the nucleus and cytoplasm of A9 cells, whereas it stains only <5% of A10 neurons proving that Hb is differentially expressed in the DA cells' groups. In the mesencephalon, Hb retains its tetrameric structure as in blood.^14^

The function of Hb in DA neurons remains unclear. We have previously shown that its overexpression in a mouse DA cell line was altering transcript levels of genes involved in oxygen (O_2_) homeostasis and oxidative phosphorylation.^12^

In this study, we provide evidence that neuronal Hb may contribute to DA cells' dysfunction in PD by interfering with pathways involved in neurodegeneration.

## Results

### Hb increases susceptibility to cell death in cellular models of PD

To investigate the role of Hb in PD, we analysed the effects of Hb overexpression upon PD-mimicking insults. We took advantage of the DA cell line iMN9D whose differentiation can be triggered by an inducible artificial system to increase the expression of Nurr1, a transcription factor involved in DA cells' differentiation and maintenance. These cells overexpress stably *α*- and *β*-chains of Hb forming the *α*_2_*β*_2_ tetramer.^12, 14^

Differentiated cells were treated with increasing concentrations of 1-methyl-4-phenylpyridinium (MPP^+^), the active ion of the neurotoxin MPTP, or of the pesticide rotenone. Both molecules induce mitochondrial dysfunction leading to caspase activation and cell death.^1, 8^ Upon their administration, we analysed the levels of cleaved Caspase-3, a marker of apoptosis, through western blotting. We then tested cell mortality with FACS and monitored the metabolic activity of viable cells with WST-1.

**Figure 1:**
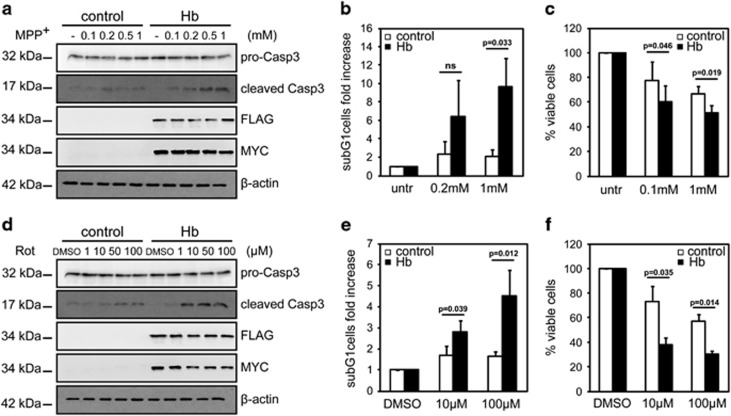
Hb increases susceptibility to cell death in cellular model of PD. Differentiated Hb cells (Hb) and control cells (control) were treated with MPP^+^ (**a–c**) or rotenone (**d–f**) at the indicated concentrations for 16 h. (**a** and **d**) Western blotting analysis of cleaved Caspase-3 (cleaved Casp3) expression. *α*- and *β*-Globins were detected with anti-FLAG and anti-MYC antibodies, respectively. For normalization, the levels of pro-Caspase-3 (pro-Casp3) were detected. *β*-Actin was used as a loading control. (*n*=3, *n*=3) (**b** and **e**) FACS analysis. Percentage of subG1 cells is expressed as fold increased relative to untreated cells, arbitrary set to 1. (*n*=3, *n*=4) (**c** and **f**) WST-1 analysis. In graph are represented the percentage of viable cells relative to untreated cells, arbitrary set to 100%. (*n*=4, *n*=3) Values are mean±S.D. Data were evaluated statistically by Student's *t*-test. Resulting *P*-values are indicated (NS=not significant)

First, we focussed our attention on differentiated cells treated with MPP^+^. Twenty-four hours after plating, doxycycline was supplemented to induce Nurr1 expression and DA cells' differentiation. MPP^+^ was added 56 h later when cells were approximately 80% confluent. After additional 16 h, cells were collected and analysed. We used MPP^+^ at the concentrations of 0.1, 0.2, 0.5 or 1 mM. Levels of cleaved Caspase-3 were higher in Hb cells than controls in a dose-dependent manner (Figure 1a). Densitometric quantification of pro-Caspase-3 and cleaved Caspase-3 are shown in Supplementary Figures S1a and c. FACS analysis demonstrated that cell death was more evident in Hb cells upon 1 mM MPP^+^ addition (Figure 1b). WST-1 assay showed that Hb cells exhibited about 20% less viability in comparison to controls upon MPP^+^ treatment (Figure 1c).

We then analysed the effects of rotenone in the same experimental settings. We used rotenone at the concentrations of 1, 10, 50 or 100 *μ*M. A higher level of cleaved Caspase-3 was evident in Hb cells when compared with controls upon rotenone treatment in a dose-dependent manner (Figure 1d). Densitometric quantification of pro-Caspase-3 and cleaved Caspase-3 are shown in Supplementary Figures S1b and d. FACS analysis showed a higher death ratio in Hb cells upon drug treatments (Figure 1e). WST-1 assay proved Hb cells presented about 30% less viability in comparison to controls upon rotenone administration (Figure 1f).

To assess whether Hb toxicity is O_2_ dependent, we generated a stable cell line overexpressing a mutant Hb (mut Hb) that is unable to bind O_2_. In detail, we mutated the proximal histidine residue (F8) of both *α*- and *β*-chains to glycine. This small residue does not allow the fifth coordination bond of the heme-Fe atom.^15, 16^ Once mutated, Hb cells were generated (Supplementary Figures S2a and b), differentiated and their mortality compared with Hb cells and controls upon PD-mimicking insults. As shown in Supplementary Figure S2c, mut Hb cells did not prevent Hb toxicity. Although there was slightly less cleaved Caspase-3 in mut Hb cells in comparison to Hb cells, we interpreted it as owing to lower Hb level in mut Hb cells (Supplementary Figure S2a).

These experiments indicate that Hb overexpression increases susceptibility to cell death in cellular models of PD with mechanisms that do not depend on O_2_ binding.

### Neurochemical intoxication increases Hb in the nucleus

**Figure 2:**
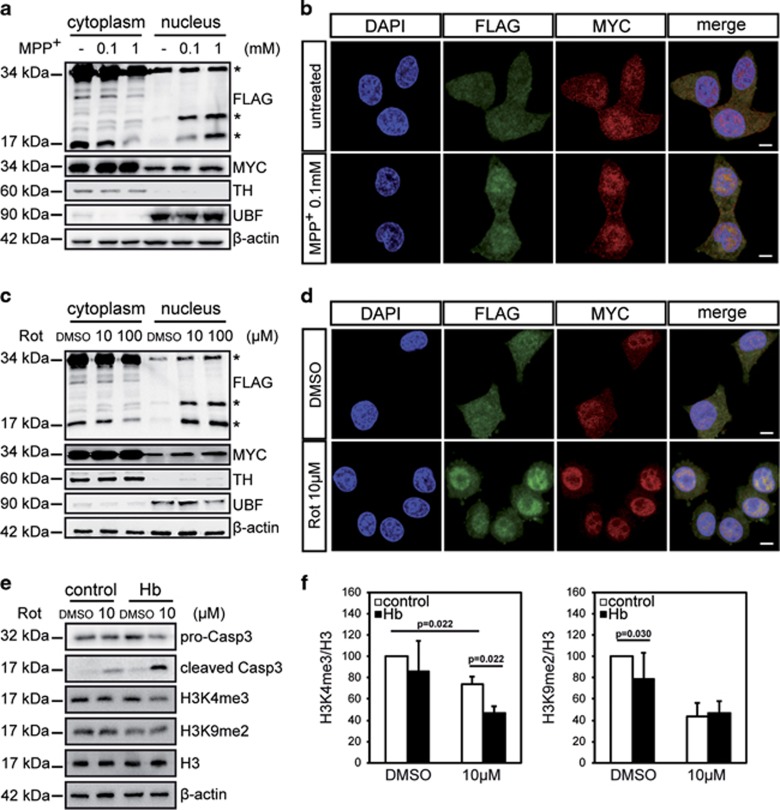
Neurochemical intoxication increases Hb in the nucleus. Differentiated Hb cells were treated with MPP^+^ (**a** and **b**) or rotenone (**c–f**) at the indicated concentrations for 16 h. (**a** and **c**) Western blotting analysis of cellular fractionation was carried out with anti-FLAG (*α*-globin) and anti-MYC (*β*-globin) antibodies. Anti-TH and anti-UBF antibodies were used to visualize specifically cytoplasm and nucleus compartment, respectively. *β*-Actin was used as a loading control. (*n*=5, *n*=4) (**b** and **d**) Double immunofluorescence was performed with anti-FLAG (*α*-globin) and anti-MYC (*β*-globin) antibodies. Nuclei were marked by DAPI (4,6-diamidino-2-phenylindole). Scale bar 5 *μ*m. (*n*=3, *n*=4) (**e**) Western blotting analysis of H3K4me3 and H3K9me2 expression. For normalization, the total levels of H3 and *β*-actin were detected. Levels of pro-Caspase-3 (pro-Casp3) and cleaved Caspase-3 (cleaved Casp3) were also monitored. (*n*=3) (**f**) Densitometric analysis of H3K4me3 and H3K9me2 expression. H3K4me3 and H3K9me2 levels were normalized to H3 and *β*-actin. Untreated control cells was used as reference and set to 100%. (*n*=3, *n*=3) Values are mean±S.D. Data were evaluated statistically by Student's *t*-test. The *P*-values were adjusted for multiple testing using the Benjamini–Hochberg method to control the false discovery rate. Resulting *P*-values are indicated

We performed cellular fractionation experiments to study the intracellular distribution of Hb upon PD-mimicking insults. In untreated conditions, *α*- and *β*-chains were present both in the cytoplasm and in the nucleus of differentiated iMN9Dcells (Figures 2a–d). Upon MPP^+^ and rotenone treatments, the levels of *α*-globin (visualized with anti-FLAG antibody) and *β*-globin (visualized with anti-MYC antibody) increased in the nucleus (Figures 2a and c). Interestingly, an *α*-globin band of about 20 kDa appeared in the nucleus, unveiling posttranslational modifications of the *α*-chain potentially associated with toxicity. Densitometric quantification of nuclear FLAG (*α*-globin) and MYC (*β*-globin) are shown in Supplementary Figures S1e–h. Mutated *α*-globin accumulated in the nucleus upon rotenone treatment as reported for wild-type Hb, indicating again that Hb-mediated toxic mechanisms do not require O_2_ binding (Supplementary Figure S2d). These results were confirmed with immunofluorescence (Figures 2b and d).

Importantly, *α*- and *β*-chains assembly was not altered upon toxic stimuli (Supplementary Figures S3a and b).

These data prove that in differentiated Hb cells the exposure to MPP^+^ and rotenone increases the amount of Hb in the nucleus and the fraction of a nuclear Hb form modified at posttranslational level.

### Hb modifies the epigenetic status of DA neurons under stress

As MPTP and rotenone have been previously shown to affect the epigenome of neurons,^17, 18^ we analysed H3 methylation status in our experimental settings (Figure 2e). The level of H3 lysine 9 dimethylation (H3K9me2) was reduced in Hb cells in comparison to control cells in untreated conditions, while treated Hb cells presented 27% less H3 lysine 4 trimethylation (H3K4me3) than controls (Figure 2f). It is well known that the H3K4me3 signature is correlated with transcription activation.^19, 20^ Among the validated targets, high levels of H3K4me3 at promoter and coding regions of the p53 gene are known to facilitate p53 transcription.^21^ By taking advantage of qRT-PCR analysis in Hb and control cells, a correlation was showed between H4K4me3 and p53 mRNA levels (Supplementary Figure S3c).

These data prove that Hb can regulate the epigenome of neurons upon stress and in untreated conditions.

### Neurochemical intoxication accumulates insoluble Hb in the nucleolus

**Figure 3:**
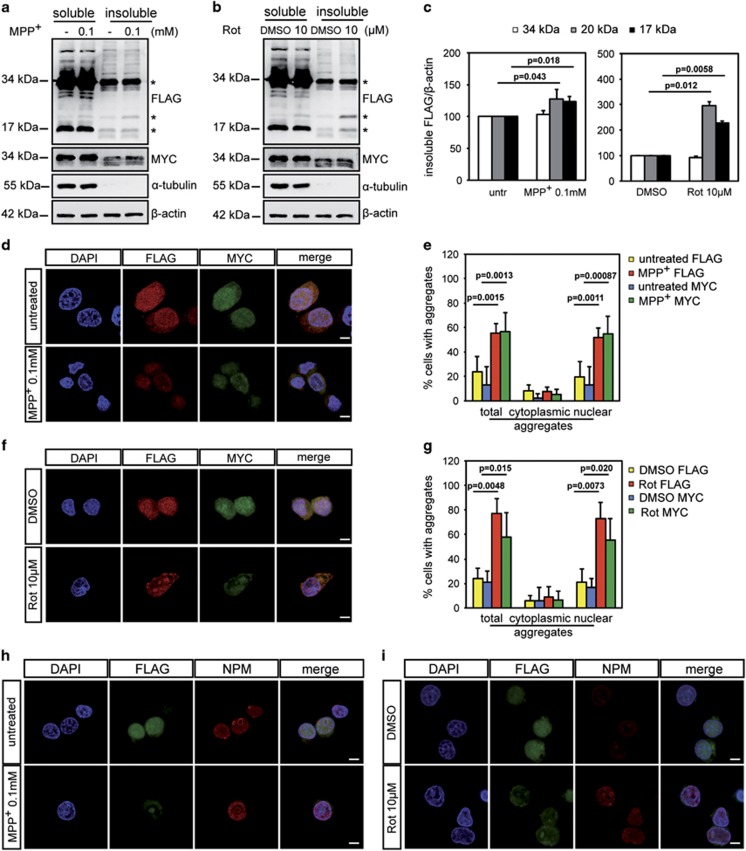
Neurochemical intoxication accumulates insoluble Hb in the nucleolus. Differentiated Hb cells were treated with MPP^+^ (**a**, **d**, **h**) or rotenone (**b**, **f**, **i**) at the indicated concentrations for 16 h. (**a** and **b**) Western blotting analysis of soluble and insoluble fractionation was carried out with anti-FLAG (*α*-globin) and anti-MYC (*β*-globin) antibodies. *α*-Tubulin was used to visualize specifically soluble fraction. *β*-Actin was used as a loading control. (FLAG *n*=3, MYC *n*=2; FLAG *n*=4, MYC *n*=2) (**c**) Densitometric analysis of insoluble FLAG (*α*-globin) level. Insoluble *α*-globin level was normalized to *β*-actin. Untreated cells was used as reference and set to 100%. (*n*=3, *n*=3) (**d** and **f**) Solubilized double immunofluorescence was performed with anti-FLAG (*α*-globin) and anti-MYC (*β*-globin) antibodies. Nuclei were marked by DAPI (4,6-diamidino-2-phenylindole). Scale bar 5 *μ*m. (*n*=3, *n*=3) (**e** and **g**) Quantification of globin chain aggregates in the cells. An average of 120 randomly chosen cells were counted for quantification of each condition. Values are expresses as a percentage relative to the total. (**h** and **i**) Solubilized double immunofluorescence was performed with anti-FLAG (*α*-globin) and anti-NPM antibodies. Nuclei were marked by DAPI. Scale bar 5 *μ*m. (*n*=2, *n*=2) Values are mean±S.D. Data were evaluated statistically by Student's *t*-test. Resulting *P*-values are indicated

As the formation of insoluble aggregates is a hallmark of PD, we tested the solubility of Hb in our experimental preparation. Figures 3a and b shows that upon treatments with MPP^+^ and rotenone *α*-chain accumulated in the insoluble portion while in untreated conditions globin chains were mainly, but not exclusively, localized in Triton X-100 soluble fractions. Furthermore, the quantification analysis of insoluble FLAG (*α*-globin)-positive bands demonstrates that there is an increase of the 17 and 20 kDa bands upon treatments (Figure 3c). Immunofluorescence analysis was then carried out after permeabilization with Triton X-100 before fixation. Although a small amount of *α*- and *β*-globin-containing aggregates were already present in untreated cells, PD-mimicking insults strongly induced Hb aggregation (Figures 3d and f) mainly in the nucleus. The quantification of Hb insoluble aggregates are shown in Figures 3e and g.

Double immunofluorescence for *α*-globins (visualized with anti-FLAG antibody) and Nucleophosmin (NPM), a specific marker for granular component of the nucleolus, proved that Hb aggregates were contained within nucleolar structures (Figures 3h and i).

These data demonstrate that PD-mimicking insults increase nucleolar aggregation of Hb.

### Hb induces nucleolar stress

**Figure 4:**
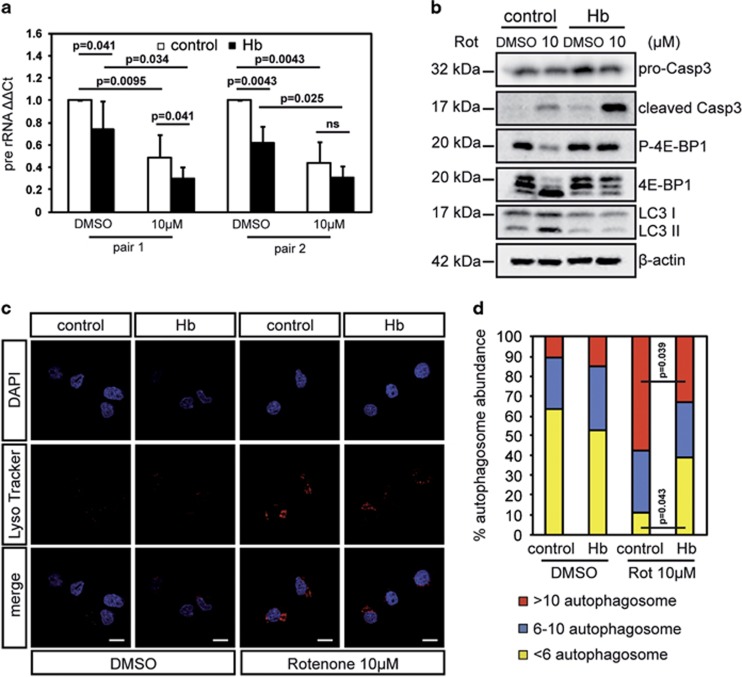
Hb induces nucleolar stress and impairs autophagy in cellular model of PD. Differentiated Hb cells (Hb) and control cells (control) were treated with rotenone at the indicated concentrations for 16 h. (**a**) qRT-PCR of pre-rRNA levels. Pre-rRNA levels were normalized to *β*-actin. Untreated control cells was used as reference and set to 1. (*n*=5) (**b**) Western blotting analysis of phosphorylated 4E-BP1 (P-4E-BP1) status and LC3 expression. For normalization, the total levels of 4E-BP1 were detected. Levels of pro-Caspase-3 (pro-Casp3) and cleaved Caspase-3 (cleaved Casp3) were also monitored. *β*-Actin was used as a loading control. (*n*=5) (**c**) LysoTracker Red staining. Nuclei were marked by DAPI (4,6-diamidino-2-phenylindole). Scale bar 10 *μ*m. (*n*=3) (**d**) Quantification of autophagosome. Ninety randomly chosen cells were counted for quantification of each condition. Values are expressed as a percentage relative to the total. Autophagy activity was defined as follows: high: >10 LysoTracker Red foci/cell; medium: 6–10 LysoTracker Red foci/cell; low: <6 LysoTracker Red foci/cell. Values are mean±S.D. Data were evaluated statistically by Student's *t*-test. The *P*-values were adjusted for multiple testing using the Benjamini–Hochberg method to control the false discovery rate. Resulting *P*-values are indicated (NS=not significant)

Given the presence of Hb aggregates in the nucleoli, we assessed the synthesis of pre-rRNA transcripts as a marker of nucleolar stress. Figure 4a shows that Hb overexpression inhibited rRNA biogenesis in untreated cells. Importantly, PD-mimicking insult decreased pre-rRNA transcripts levels both in Hb cells and controls although with a statistically significant stronger effect on Hb cells.

These data indicate that Hb overexpression increases nucleolar stress affecting rRNA biosynthesis.

### Hb impairs autophagy induced by neurochemical insults

Autophagy is a crucial homeostatic response of DA cells to neurochemical intoxication and its pharmacological induction with rapamycin protects cellular and animal models of PD.^8, 9^ To investigate the effects of Hb overexpression, we monitored the phosphorylation status of the eukaryotic initiation factor 4E binding protein 1 (4E-BP1), a direct target of mTOR,^22^ showing that in Hb cells the phosphorylation of 4E-BP1 was stable while reduced in control cells, as expected (Figure 4b).

These results indicate that Hb expression inhibits downregulation of mTOR activity by rotenone treatment. Densitometric quantification of P-4EB-P1 is shown in Supplementary Figure S1l.

We then monitored the induction of LC3II as a marker of autophagosome formation and therefore of autophagic activity. Although rotenone treatment triggered increased levels of LC3II in control cells, in Hb cells it failed to do so (Figure 4b). Densitometric quantification of LC3II is shown in Supplementary Figure S1m. To further assess the impact of Hb on autophagy, neurons were stained with LysoTracker Red (Figure 4c), which labels acidic organelles, including autophagosomes in living cells. Upon rotenone treatment, the quantification of LysoTracker Red-positive foci per cell demonstrated in Hb cells the inhibition of the prominent rise of autophagosome density observed in controls (Figure 4d).

These data demonstrate that Hb overexpression is associated with an impairment of autophagy induction by preserving mTOR activity.

### AAV9-mediated delivery of Hb in SNpc inhibits improvement of motor performance and triggers Hb aggregates in DA cells

To assess the role of Hb in DA neurons *in vivo*, we overexpressed Hb in SNpc of mouse brain. Eight-week-old male C57Bl/6 mice were injected with a mixture of AAV9-2xFLAG-*α*-globin and AAV9-*β*-globin-MYC (indicated as AAV9-Hb) or with AAV9-control into SNpc of mouse brain. The contralateral SNpc remained uninjected. Supplementary Figure S4 shows the characterization of AAV9 vector injection in SNpc of mouse brain. Double immunofluorescence for Hb and the specific DA marker tyrosine hydroxylase (TH) revealed that AAV9 vectors were efficiently transduced in DA neurons in the ipsilateral side (Supplementary Figures S4d and e). Furthermore, double immunofluorescence for tagged *α*- and *β*-globin demonstrated their co-localization in DA cells, confirming the efficiency of co-infection (Supplementary Figure S4g).

**Figure 5:**
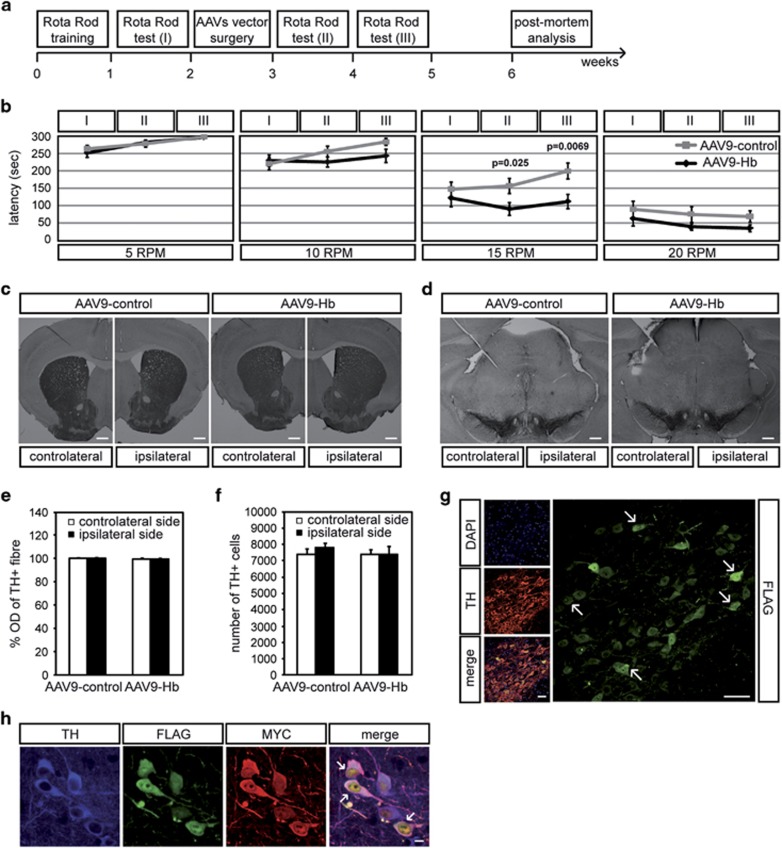
AAV9-mediated delivery of Hb in SNpc inhibits improvement of motor performance and triggers Hb aggregates in DA cells. (**a**) Scheme of experimental settings. (**b**) Latency in rotarod test in AAV9-control (*n*=18) and AAV9-Hb (*n*=19) mice at 5, 10, 15 and 20 r.p.m. (**c**) Immunohistochemistry of coronal sections of AAV9-control and AAV9-Hb mouse brain. TH^+^ fibres in the striatum were stained with anti-TH antibody. Scale bar 800 *μ*m. (**d**) Immunohistochemistry of coronal sections of AAV9-Hb and AAV9-control mouse brain. SN was stained with anti-TH antibody. Scale bar 200 *μ*m. (**e**) Densitometric analysis of TH^+^ fibres in AAV9-Hb (*n*=18) and AAV9-control (*n*=18) mice. Values are expresses as a percentage relative to the contralateral side of AAV9-control mice, arbitrary set to 100%. (**f**) Quantitative analysis of TH^+^ cells number in AAV9-Hb (*n*=16) and AAV9-control (*n*=15) mice. Values are expressed as the number of total TH^+^ cells relative to the contralateral side of AAV9-control mice. (**g**) Immunohistochemistry of coronal sections of AAV9-Hb mouse brain. SN was stained with anti-TH antibody. Infected cells were stained with anti-FLAG (*α*-globin) antibody. Arrow indicates *α*-globin aggregates. Nuclei were visualized with DAPI (4,6-diamidino-2-phenylindole). Scale bar 50 *μ*m. (**h**) Immunohistochemistry of coronal sections of AAV9-Hb mouse brain. SN was stained with anti-TH antibody. Infected cells were stained with anti-FLAG (*α*-globin) and anti-MYC (*β*-globin) antibodies. Arrow indicates globin aggregates. Scale bar 10 *μ*m. All data are represented as mean±S.E.M. Data were evaluated statistically by one-way analysis of variance. Resulting *P*-values are indicated

To determine Hb overexpression effects on DA neurons, we monitored mice motor coordination using rotarod test. We assayed AAV9-Hb and AAV9-control mice at the indicated weeks (Figure 5a). Data in Figure 5b show the mice latency, defined as the amount of time the mice stayed on the rotating rod at different r.p.m. No differences were observed in performance between AAV9-Hb and AAV9-control mice at all four speeds before AAV9 vector injection. During the time of the experiment, there was no group difference in performance at 5 and 10 r.p.m. Strikingly, when the difficulty of the test was increased (15 r.p.m.) we observed a statistical difference between AAV9-Hb- and AAV9-control-injected mice unveiling changes in locomotor activity that increased with time: AAV9-Hb (I) 123±25, (II) 91±18, (III) 112±20; AAV9-control (I) 148±21, (II) 157±22, (III) 200±23. Although AAV9-control mice improved their performance, AAV9-Hb mice failed to do so. Finally, both groups had an unsuitable performance at 20 r.p.m.

These data demonstrate that AAV9-mediated Hb expression in SNpc inhibits improvement in performance associated with motor learning. Recent evidence suggests that DA neurotrasmission is linked to cognitive function, in particular DA depletion impairs motor learning.^23^ Consequently, we performed HPLC on differentiated Hb and control cells to measure DA content. As shown in Supplementary Figure S5, DA level is significantly reduced by about 50% in Hb cells in comparison to controls.

Four weeks after AAV9 vector injection, mice were killed and brain tissues were processed for immunohistochemical analysis. We first performed a densitometric analysis of TH^+^ fibres in the striatum, direct target of DA innervation from the SN. We observed that Hb overexpression did not alter TH^+^ fibres comparing AAV9-Hb and AAV9-control mice (Figures 5c and e). We then quantified DA neurons counting A9 cells in SNpc with a computer-assisted stereological assessment tool. We first proved that the injection itself did not cause damage, as the number of TH^+^ cells in the ipsilateral and contralateral sides of AAV9-control mice were equal (Figures 5d and f). We then showed that the total number of DA neurons was not altered by Hb overexpression. Interestingly, Hb-overexpressing TH^+^ cells presented cytoplasmic and nuclear *α*-globin aggregates (Figure 5g). The majority of these structures were immunoreactive for both tagged-globin proteins (Figure 5h).

These data suggest that Hb overexpression does not alter DA cells' anatomy but triggers aggregate formation in TH^+^ neurons.

## Discussion

Globins are present in all kingdoms of living organisms where they display a variety of functions, including O_2_ sensing, transport and storage, the synthesis and scavenging of reactive nitrogen and oxygen species and heme-based catalysis.^24^ In the past decade, Hb *α*- and *β*-chains have been found expressed in a plethora of nonerythroid cells.^24^

Several reports have shown regulated expression of *α*- and *β*-chain transcripts as well as Hb immunoreactivity in the mammalian brain, including in neurons of the mesencephalon, cortex (CTX) and hippocampus^13, 25, 26, 27^ as well as in oligodendrocytes and selective astrocytes.^12, 28^

Recent evidences suggest the involvement of Hb in neurodegenerative diseases. First, an increase of Hb expression has been observed in neurons of aging brains in rodents and human.^29, 30^ Hb was found highly expressed in the neurons and glia in Alzheimer's disease (AD) brains, in APP/PS1 transgenic AD mice models and in the cerebrospinal fluid of AD patients.^30^ Hb co-localized with senile plaques and vascular amyloid deposits in AD while *α*- and *β*-chains were shown to interact with A*β* oligomer in AD brain homogenates promoting A*β* oligomer formation.^30^ These results have led to the intriguing hypothesis that Hb may have an important role in the association between cerebral vascular diseases and AD.^31^

Transcriptome profiling showed that *α*- and *β*-globins were the two most induced transcripts in the white matter of patients suffering from multiple system atrophy.^32^ By contrast, they were the two most downregulated transcripts in the brains from bovine spongiform encephalopathy-infected cynomolgus macaques, a model for human prion disorders.^33^

Several intriguing observations suggest Hb as a potential player in PD.^34^ Its distribution in mesencephalic DA neurons enlists Hb into the small number of genes whose expression correlates with cells' vulnerability.^2, 12^ *α*- and *β*-Chains mRNAs were found strongly downregulated in DA neurons of rats treated with low doses of rotenone.^35^ Intriguingly, they were upregulated in *post mortem* PD brains.^36^ Furthermore, Hb and *α*-synuclein complex was recently found in brain tissue and red blood cells of aging cynomolgus monkeys.^37^

We previously showed that overexpression of *α*- and *β*-chains in iMN9D cells altered the expression of genes involved in mitochondrial activity.^12^ Since then, Hb has been found accumulated in the mitochondria of human SN neurons and frontal CTX cells from *post mortem* PD brains.^38, 39^ Here we have demonstrated that Hb overexpression increases susceptibility to cell death in differentiated iMN9D cells exposed to MPP^+^ and rotenone. Both drugs are known to inhibit complex I of the mitochondrial electron transport chain provoking a decrease of the ATP reserve.^7^ Interestingly, Hb accumulates in the mitochondria of *post mortem* brain tissues of other diseases such as multiple sclerosis (MS), where it interacts with several mitochondrial proteins, including ATP synthase subunits.^40^

In this context, here we show for the first time that Hb is able to interfere with pathways crucial to PD pathogenesis such as DA content, nucleolar function, autophagy and epigenetic remodelling. Importantly, Hb forms insoluble aggregates in DA neurons *in vitro* and *in vivo*. Although the majority of these phenotypes are already present in untreated cells, they are strongly induced upon neurochemical intoxication.

The formation of detergent-insoluble aggregates is considered one of the hallmarks of PD. Hb aggregates are induced by neurochemical intoxication *in vitro* and they accumulate upon *α*- and *β*-chain overexpression in DA neurons of the SN *in vivo*. Aggregates are formed prevalently in the nucleolus and are associated with a posttranslational modification of the *α*-chain present in the insoluble fraction. Further work is needed to assess its identity and role in Hb toxicity.

Hb aggregates are localized in the nucleoli *in vitro*. The main function of the nucleolus is to coordinate ribosome biogenesis although it is increasingly evident that it acts as a response centre to various insults, including oxidative and proteotoxic stresses.^41^ Nucleoli have been found essential to the survival of DA neurons *in vivo*^11^ while their structure and function are altered in *post mortem* PD brains.^42, 43^ Interestingly, neurotoxic stimuli inhibited rRNA synthesis and impaired rRNA biogenesis.^11^ In this study, we show that Hb overexpression decreases pre-rRNA transcription inducing nucleolar stress upon intoxication and in untreated conditions. Although in the short-term this is considered a defence mechanism to limit energy squander preserving cell survival, protracted downregulation of rRNA transcription results in severe cellular damage and cell death.^44^ In this context, it has been recently demonstrated that the ablation of the RNA polymerase I-specific transcription initiation factor IA causes disruption of nucleoli and a transient pro-survival response, involving the inhibition of mTOR signaling and the activation of autophagy.^45^

Dysregulation of the autophagic pathway has been observed in human *post mortem* PD brains and in animal models while genes mutated in familial PD are involved in its regulation.^46^ Importantly, autophagy enhanced by rapamycin protects against cell death caused by MPTP and rotenone.^8, 47^ An intriguing relationship may thus be hypothesized between Hb toxicity and its ability to inhibit mTOR impairing autophagy.

The role of epigenetics in PD is under intense scrutiny. Dopamine depletion is associated with a reduction in histone H3K4me3, whereas treatment with MPTP and rotenone induces H3 acetylation.^18, 48^ The expression of epigenetic modifiers is dysregulated in the blood of living PD patients.^18^ Interestingly, *β*-globin interacts with H3 and histone lysine demethylase in *post mortem* MS CTX,^40^ suggesting that it may regulate H3 methylation status. It is therefore intriguing that upon PD-mimicking insults we observed an increase of Hb in the nucleus and a concomitant decrease of H3K4me3.

We then studied the effects of Hb overexpression *in vivo*. Two striking phenotypes were observed in the timeframe of the experiment (1 month). As *in vitro*, aggregates are present in the nucleus of TH^+^ cells. In the large majority of cases, they contain both *α*- and *β*-chains. Most importantly, we observed a significant difference in motor behaviour between AAV9-Hb mice and AAV9-controls. To our knowledge, this is the first experimental evidence of behavioural effects of neuronal Hb. As it is well known that changes in DA neurotransmission result in motor skill learning deficit^23^ and that we observed a reduction of DA content owing to Hb overexpression, we speculate that Hb may influence the physiological status of DA neurons leading to a dysregulation of DA neurotransmission. No changes in DA histology were found considering densitometric analysis of TH^+^ fibres in the striatum and the number of total TH^+^ neurons in SN. Although genetic inhibition of rRNA biogenesis requires several months to trigger DA cells' degeneration, AAV9-Hb mice with motor skill learning deficit were analysed 1 month upon AAVs injection, opening the possibility that neuronal damage may become evident at later time.

Although our data prove that neuronal Hb regulates pathways involved in PD pathogenesis and potentially DA activity *in vivo*, its function remains unclear. By replacing the highly conserved proximal histidine F8 residue with a glycine, mutated Hb presents a low affinity for the heme group and a loss of its allosteric properties.^15, 16^ In these conditions, we observed that mutated Hb confers susceptibility to neurotoxic stimuli as wild-type Hb, suggesting that Hb toxicity does not depend on O_2_ binding.

Overall, we hypothesize that neuronal Hb acts as a sensor of the energy status of neurons. By linking ATP concentration and mitochondrial function to mTOR activity, it may regulate cells' stress response acting on ribosome biogenesis, autophagy, the epigenome and, ultimately, DA cells' neurotransmission.

Although it is unclear how Hb expression is regulated in nonerythroid cells, A9 neurons present the expression of GATA family members^12, 49^ that are the major Hb transcriptional regulators. In this context, any environmental or genetic conditions that modify Hb levels in A9 cells may affect neuronal susceptibility. Interestingly, Hb chains have been found highly induced by low iron diet in the ventral midbrain of adult mice, implicating them in brain iron homeostasis, a pathway involved in PD as well.^50^ On the other hand, it will be interesting to assess the relationship, if any, of Hb levels in blood and DA cells in normal and pathological conditions of hematological origin, such as thalassemia. Recent reports^51, 52^ associate blood Hb levels and PD, suggesting that high Hb is linked to an increase risk of PD.

Our results support a model where Hb levels in A9 neurons may be associated with cellular susceptibility in PD, positioning Hb in the list of potential PD modifiers that deserve further attention.

## Materials and methods

### Generation of stable cell line

From pBudCE4.1-*β*-globin-MYC-IRES-eGFP, 2xFLAG-*α*-globin, as previously described,^12^ we generated a mutated mouse Hb, that we called pBUD-mut *β*-globin-MYC-IRES-eGFP, 2xFLAG-mut *α*-globin. In detail, the proximal hystidines of *α*- and *β*-chains were mutated into a glycine (respectively, His87Gly and His92Gly). We used the QuikChange Site-Directed Mutagenesis Kit (Stratagene, San Diego, CA, USA) according to the manufacturer's instructions. The list of primers used for mutagenesis is in Supplementary Table S1. iMN9D cell line was transfected with pBUD-mut *β*-globin-MYC-IRES-eGFP, 2xFLAG-mut *α*-globin using Lipofectamine 2000 (Life Technologies, Carlsbad, CA, USA) according to the manufacturer's instructions. After 24 h of transfection, cells were collected. A cell strainer with 70 *μ*m nylon mesh (BD Falcon, Franklin Lakes, NJ, USA) was used to obtain a single-cell suspension before sorting. 7-AAD (Beckman-Coulter, Brea, CA, USA) was added to the cell suspension to exclude dead cells. A high-speed cell sorter (MoFlo, Brea, CA, USA) was used to sort subpopulation of cells expressing green fluorescent protein (GFP). Sorting parameters used were described previously.^12^ After sorting, cells were re-plated, and 48 h later, 300 *μ*g/ml zeocyn (Life Technologies) was added for selection.

### Cell cultures and treatments

We used MN9D-Nurr1^Tet-on^ (iMN9D) cell line stably transfected with pBUD-IRES-eGFP (that we called control cells) or with pBUD-*β*-globin-MYC IRES-eGFP, 2xFLAG-*α*-globin (that we called Hb cells), as previously described,^12^ or with pBUD-mut *β*-globin-MYC-IRES-eGFP, 2xFLAG-mut *α*-globin (that we called mut Hb cells). Cells were maintained in culture using DMEM/F12 medium (Life Technologies) supplemented with 10% fetal bovine serum (Sigma-Aldrich, St. Louis, MO, USA), 100 *μ*g/ml penicillin (Sigma-Aldrich), 100 *μ*g/ml streptomycin (Sigma-Aldrich), 300 *μ*g/ml neomycin (Life Technologies) selection and 150 *μ*g/ml zeocyn (Life Technologies) selection at 37 °C in a humidified CO_2_ incubator. Nurr1 expression in iMN9D cell lines were induced by addition of 3 *μ*g/ml doxycycline (Sigma-Aldrich) to the culture medium every 48 h. Cells were grown as above except that the 10% fetal bovine serum was changed to 5%.^53^ For treatments, the following reagents were used: MPP^+^ (Sigma-Aldrich) and rotenone (Sigma-Aldrich) for 16 h at different concentrations.

### Generation and purification of recombinant AAVs

We used AAV serotype 9 for our experiments because of its high expression level and its brain tropism.^54^ The fragments corresponding to 2xFLAG-*α*-globin and *β*-globin-MYC were cloned into the pAAV-MCS vector (Agilent Technologies, Santa Clara, CA, USA), which was used to produce recombinant AAV vectors. In addition, we use pAAV-MCS vector as control. AAV of serotype 9 were generated in HEK 293 T cells, using a triple-plasmid co-transfection for packaging. Viral stocks were obtained by CsCl_2_ gradient centrifugation. Titration of AAV viral particles was performed by real-time PCR quantification of the number of viral genomes. The viral preparations had the following titres: AAV9-2xFLAG-*α*-globin 7 × 10^13^ viral genome particles/ml, AAV9-*β*-globin-MYC 8.8 × 10^13^ viral genome particles/ml and AAV9-control 7.5 × 10^12^ viral genome particles/ml.

### Animals

All animal experiments were performed in accordance with European guidelines for animal care and following Italian Board of Health permissions (DM 2/2012-B, 9 January 2012). Mice were housed and bred in SISSA animal facility, with 12 h dark/light cycles and controlled temperature and humidity. Food and water were provided *ad libitum*.

### Rotarod test

To measure locomotor activity, mice were tested with a rotarod apparatus with automatic timers and falling sensors (Crisel Instruments, Roma, Italy) at the indicated number of weeks during the light phase of the 12 h light/12 h dark cycle. Mice were first trained on the rotarod for 5 consecutive days. Animals that stayed on the rod for 300 s at 5 r.p.m. during training were selected and were randomly assigned to two groups. Mice were tested for four trials (maximum of 300 s/trial) at 5, 10, 15 and 20 r.p.m. For each trial, the time each mouse remained on the rotating rod was recorded and the best three trials were averaged as a measure of motor performance.

### Stereotaxic surgery

The stereotaxic procedure followed were according to Cetin *et al.*^55^ Adult (8 week old) male C57Bl/6 mice weighing 25 g±3 g were used for experiments. Mice were preanaesthetized with Tramadolo i.p. (30 mg/kg body weight) and anaesthetized with a mixture of Xylazina (15 mg/kg body weight) and Zoletil (15 mg/kg body weight) i.p. A stereotaxic injection of 3 *μ*l of viral vector suspension (AAV9-control or a mixture of AAV9-2xFLAG-*α*-globin and AAV9-*β*-globin-MYC, that we called AAV9-Hb) was delivered to the left SNpc. The coordinates were: anterior/posterior (A/P) −3.2 mm from bregma, medio/lateral (M/L) −1.2 mm from bregma and dorso/ventral (D/V) −4.5 mm from the dura. The coordinates used were calculated according to the Franklin and Paxinos Stereotaxic Mouse Atlas. The injection rate was 0.2 *μ*l/30 s using a 10 *μ*l syringe with 26 gauge needle (SGE, Ringwood, VIC, Australia).

### Tissue collection and processing

At the indicated number of weeks after injection of AAVs into SNpc, the animals were killed. Following induction of deep anaesthesia with an overdose of a mixture of Xylazina and Zoletil, the animals were intensively perfused transcardially with PBS 1 ×. For biochemical analysis, SN, striatum and CTX were dissected and immediately frozen in liquid nitrogen and stored at −80 °C, pending analyses. For immunohistochemical analysis, after the intensively transcardially perfusion with PBS 1 ×, animals were perfused with 4% paraformaldehyde diluted in PBS 1 ×. Brains were immediately dissected with the Mouse Brain Matrix instrument (Protech International Inc., San Antonio, TX, USA) and separated into two pieces, the anterior part containing the striatum and the posterior one containing SN. Brains were postfixed in 4% paraformaldehyde for 1 h at 4 °C. The regions containing the striatum and SN were cut in 40 *μ*m free-floating slides with a vibratome (Vibratome Series 1000 Sectioning System, Technical Products International, St. Louis, MO, USA). Four consecutive series were collected in order to represent the whole area of interest.

### PCR and quantitative RT-PCR (qRT-PCR)

Total RNA was extracted from cell and mouse tissue samples using the TRIzol reagent (Life Technologies) following the manufacturer's instructions. Mouse tissues were homogenized using a glass-Teflon (Thermo Scientific, Waltham, MA, USA). A fraction of the total RNA samples were subjected to DNase I treatment (Life Technologies) at 37 °C for 1 h and the sample was then purified on RNAeasy mini kit columns (Qiagen, Hilden, Germany). The final quality of RNA sample was tested on agarose gel. Single-strand cDNA was obtained from 1 *μ*g of purified RNA using the iSCRIPT cDNA Synthesis Kit (Bio-Rad, Hercules, CA, USA) according to the manufacturer's instructions. To amplify transcripts, Ex Taq DNA polymerase (Takara Bio Inc., Kasatsu, Japan) was used. qRT-PCR was performed in triplicate using SYBER-Green PCR Master Mix (Bio-Rad) and an iCycler IQ Real Time PCR System (Bio-Rad). Relative gene expression was calculated with ΔΔCt method. The complete list of primers used for PCR and RT-qPCR is in Supplementary Table S1. All amplicons were sequenced.

### Western blotting

Proteins derived from adherent and floating cells were collected, washed intensely with PBS 1 × and lysed in SDS sample buffer 2 ×. Proteins derived from mouse tissue were obtained using TRIzol reagent (Life Technologies) following the manufacturer's instructions. Proteins were separated in 10–17% SDS polyacrylamide gel as needed. After the separation on gel, proteins were transferred to nitrocellulose membrane (GE Healthcare Life Science, Buckinghamshire, UK). Membrane was blocked with 5% nonfat milk in TBST solution (TBS and 0.1% Tween20) and then incubated with primary antibodies overnight at 4 °C or at room temperature for 2 h. The following antibodies were used: anti-Caspase-3 1:1000 (Cell Signaling, Danvers, MA, USA), anti-cleaved Caspase-3 1:1000 (Cell Signaling), anti-FLAG 1:2000 (Sigma-Aldrich), anti-MYC 1:2000 (Cell Signaling), anti-*β*-actin 1:10000 (Sigma-Aldrich), anti-TH 1:1000 (Sigma-Aldrich), anti-UBF 1:1000 (Santa Cruz, Dallas, TX, USA), anti-H3K4me3 1:5000 (Millipore, Billerica, MA, USA), anti-H3K9me2 1:5000 (Abcam, Cambridge, UK), anti-H3 1:10000 (Cell Signaling), anti-*α*-tubulin 1:100 (purified from immunized rabbits), P-4E-BP1 1:1000 (Cell Signaling), 4E-BP1 1:1000 (Cell Signaling), anti-LC3b 1:1000 (Cell Signaling), anti-Hemoglobin 1:1000 (MP Biomedicals, Santa Ana, CA, USA) and anti-GFP 1:1000 (Life Technologies). For development, secondary antibodies conjugated with horseradish peroxidase (Dako, Glostrup, Denmark) were used in combination with ECL reagent (GE Healthcare Life Science). Image acquisition was performed using the Alliance 4.7 software (UVITEC, Cambridge, UK). Quantification of protein bands from western blotting scans was performed with the ImageJ Software (National Institute of Health, Bethesda, MD, USA).

### Coimmunoprecipitation

Cells were lysed in immunoprecipitation buffer (300 mM NaCl, 50 mM Tris pH 7.5, 1% Nonidet P-40, 10% glycerol), supplemented with protease inhibitor mixture (Roche Diagnostics, Basel, Switzerland) for 30 min at 4 °C. Lysates were cleared at 15 000 × *g* for 20 min. Cell lysates were incubated with anti-FLAG agarose beads (Sigma-Aldrich). After washing, immunoprecipitated proteins were eluted with SDS sample buffer 2 ×, boiled and analyzed by western blotting.

### Cellular fractionation

Nucleo-cytoplasmic separation was performed using the Nucleo-Cytoplasmic Separation Kit (Norgen Biotek Corp., Thorold, ON, Canada) according to the manufacturer's instruction. The effectiveness of cellular separation was controlled with cytoplasmic and nuclear markers TH and UBF, respectively.

### Detergent-solubility fractionation

Detergent solubility was performed as previously described.^56^ In detail, cells were harvested in buffer containing 50 mM Tris-HCl pH 7.4, 175 mM NaCl, 5 mM EDTA pH 8.0 supplemented with protease inhibitor mixture (Roche Diagnostics). Cells were lysed once through a 30 gauge needle and sonicated for 10 s. After the addition of Triton X-100 (final concentration 1%), lysates were incubated for 30 min on ice and centrifugated at 15 000 × *g* for 1 h at 4 °C in order to separate the Triton X-100 soluble (supernatant) and insoluble (pellet) fractions. The pellet was dissolved in 2% SDS-containing lysis buffer and sonicated for 10 s. The effectiveness of cellular separation was controlled with *α*-tubulin, a marker of the soluble fraction.

### Fluorescence-activated cell sorter analysis

For FACS analysis, both adherent and floating cells were collected and fixed in ice-cold 70% ethanol. After rehydration, cells were suspended in PBS+0.1% NP40+200 *μ*g/ml RNaseA and treated with 40 *μ*g/ml propidium iodide and analyzed on a flow cytometer (FACSCalibur, BD Biosciences, Franklin Lakes, NJ, USA). At least 10^4^ GFP-positive cells were analysed in each acquisition. FACS data were processed using the FlowJo software (Tree Star Inc., Ashland, OR, USA) and cell cycle profiles were determined using the Watson pragmatic mode.

### WST-1 analysis

Cell viability was assayed using WST-1 reagent (Roche Diagnostics), according to the manufacturer's protocol. In detail, absorbance was measured on a microplate ELISA reader (Thermo Scientific) at a 450 and 620 nm detection wavelength. Normalized WST was calculated as follows: WST(%)=((A_450nm_–A_620 nm_ of treated sample−A_450 nm_–A_620 nm_ of blanck)/(A_450 nm_–A_620 nm_ of untreated sample−A_450nm_–A_620nm_ of blanck)) × 100. Blank represents wells containing culture medium only.

### Immunocytochemistry

Cells were fixed in 4% paraformaldehyde for 10 min, then washed with PBS 1 ×, treated with 0.1 M glycine for 4 min in PBS 1 × and permeabilized with 0.1% Triton X-100 in PBS 1 × for 4 min. After washing with PBS 1 × and blocking with 0.2% BSA, 1% NGS, 0.1% Triton X-100 in PBS 1 ×, cells were incubated with primary antibodies diluted in blocking solution for 90 min at room temperature. After washes in PBS 1 ×, cells were incubated with labelled secondary antibodies for 60 min. For nuclear staining, cells were incubated with 1 *μ*g/ml DAPI for 5 min. Cells were washed and mounted with Vectashield mounting medium (Vector Laboratories, Burlingame, CA, USA). The following antibodies were used: anti-FLAG 1:1000 (Sigma-Aldrich), anti-MYC 1:2000 (Cell Signaling) and anti-NPM 1:100 (Invitrogen, Carlsbad, CA, USA). For detection, Alexa Fluor-488, -594 or -405 (Life Technologies) antibodies were used. To detect insoluble Hb, immunocytochemistry was performed as previously described.^42^ All images were collected using confocal microscopes (LEICA TCS SP2, Wetzlar, Germany and Nikon D-Eclipse C1, Tokyo, Japan). Using the Volocity 3D Image Analysis Software (PerkinElmer, Waltham, MA, USA), aggregates per cell were counted. Values were represented as percentage of the total.

### LysoTracker staining

Cells were stained without fixation with 50 nM LysoTracker Red (Molecular Probes, Eugene, OR, USA) in growth medium for 1 h. Stained cells were immediately fixed with 4% paraformaldeide and follow a standard immunofluorescence protocol. LysoTracker staining were visualized with a confocal microscope (Nikon D-Eclipse C1). Using Volocity 3D Image Analysis Software (PerkinElmer), autophagosomes per cell were counted. Autophagy activity was defined as follows:^57^ high: >10 autophagosomes/cell; medium: 6–10 autophagosomes/cell; and low: <6 autophagosomes/cell. Values were represented as percentage of the total.

### Immunohistochemistry

For immunohistochemistry, a standard avidin–biotin ABC procedure was performed on free-floating slides. Endogenous peroxidase activity was first quenched by a 10 min incubation in 10% methanol with 3% hydrogen peroxide in PBS 1 × , followed by incubation in 10% NGS, 1% BSA and 1% Fish gelatin in PBS 1 × for 1 h. The primary and secondary antibodies were diluted in 1% BSA, 0.3% Triton X-100 and 0.1% Fish gelatin in PBS 1 ×. Incubation with primary antibodies 1:1000 anti-TH (Sigma-Aldrich) was performed overnight at RT. Incubation with 1:200 biotinylated secondary antibody (Santa Cruz) was performed for 1 h at RT, followed by 1 h incubation in avidin–biotin–peroxidase solution (ABC Kit, Vector Laboratories). The staining was visualized using 3,3′-diaminobenzidine (Sigma-Aldrich) as a chromogen for 2 min. The sections were then mounted on slides (Thermo Scientific SuperFrost Plus), dehydrated in ascending alcohol concentrations, cleared in xylene and mounted with Eukitt mounting medium (O. Kindler GmbH, Freiburg, Germany). All images were collected in bright-field (LEICA DM6000).

For fluorescent immunohistochemistry, free-floating slides were treated with 0.1 M glycine for 5 min in PBS 1 × and then with 1% SDS in PBS 1 × for 1 min at RT. Slides were blocked with 10% NGS, 1% BSA and 1% Fish gelatin in PBS 1 × for 1 h at RT. The antibodies were diluted in 1% BSA, 0.3% Triton X-100 and 0.1% Fish gelatin in PBS 1 ×. For double immunoflurescence, incubation with primary antibodies was performed overnight at RT and incubation with 1:500 Alexa fluor-conjugated secondary antibodies (Life Technologie) was performed for 2 h at RT. Nuclei were labelled with 1 *μ*g/ml DAPI. For triple immunofluorescence, incubation with primary antibodies was performed overnight at RT, incubation with 1:500 Alexa fluor-conjugated secondary antibodies (Life Technologies) and 1:100 biotin-labelled secondary antibody (Sigma-Aldrich) was performed for 2 h at RT, followed by 1 h incubation in 1:100 streptavidin, Marina Blue conjugate (Life Technologies). Slides were mounted with mounting medium for fluorescence Vectashield (Vector Laboratories). The following primary antibodies were used: anti-TH 1:1000 (Sigma-Aldrich or Millipore), anti-FLAG 1:100 (Sigma-Aldrich), anti-MYC 1:100 (Cell Signaling) and anti-Hemoglobin 1:1000 (MP Biomedicals). For detection, Alexa fluor-488 or -594 (Life Technologies) were used. All images were collected using confocal microscopes (LEICA TCS SP2).

### Dopamine determination by HPLC

Cells were lysed in 200 *μ*l of 0.1 M perchloric acid, sonicated and spun in a microcentrifuge at 10 000 × *g* for 10 min at 4 °C. The supernatant was transferred in ultra-free microcentrifuge tubes (Millipore) and spun at 10 000 × *g* for 5 min at 4 °C. Samples (11 *μ*l) were injected into the HPLC apparatus. Measurements of dopamine were made by HPLC with an electrochemical detection system (ALEXYS LC-EC, Antec Leyden BV, Zoeterwoude, The Netherlands) equipped with a reverse-phase column (3-*μ*m particles, ALB-215 C18, 1 × 150 mm, Antec Leyden BV) at a flow rate of 50 *μ*l/min and electrochemically detected by a 0.7 mm glass carbon electrode (VT-03, Antec Leyden BV). The mobile phase contained 50 mM H_3_PO_4_, 50 mM citric acid, 8 mM KCl, 0.1 mM EDTA, 400 mg/l octanesulfonic acid sodium salt and 8% (vol/vol) acetonitrile (pH 3.0).

### Determination of striatal DA density

DA innervation in the striatum was estimated via optical density (OD) determination of immunostaining using Leica DM6000 microscope. Briefly, striatal sections were immunostained with anti-TH antibody. Scans of three different section were acquired corresponding to the rostral, intermediate and caudal portion of the striatum. Using the ImageJ Software (National Institute of Health), the region of interest on the section was outlined and the mean OD value for the region was determined. A background value, corresponding to a region devoid of immunoreactivity (i.e., corpus callosum), was subtracted from each determination. The OD values were calculated as arbitrary units and were expressed as a percentage relative to the contralateral side of control-injected mice.

### Quantification of DA neurons in the SNpc

One out of the four series of 40 *μ*m freefloating slides were immunostained with anti-TH antibody. SNpc was outlined under a low magnification objective (× 4) following landmarks from the Franklin and Paxinos Mouse Atlas and was analyzed under a × 100 objective of an Olympus BH2 microscope (Tokyo, Japan). Cells were counted throughout the whole SNpc area and through the entire section thickness. Quantification of TH^+^ cells was performed according to a stereological approach using the Olympus CAST-Grid system (Olympus Denmark A/S, Albertslund, Denmark). TH^**+**^ cells were counted by a systematically random sampling scheme using approximately 160 × 160 *μ*m^2^ optical dissectors. All counts were performed blind to the experimental status of the animals. The orientation for each brain was determined by marking one side of each brain by performing an incision through the contralateral side of the brain. The TH^+^ cell values were expressed relative to the contralateral side of control-injected mice.

### Statistical analysis

All data were obtained by at least three independent experiments. Data represent the mean±S.D.; each group was compared individually with the reference control group using Student's *t*-test (Microsoft Excel software, Microsoft, Redmond, WA, USA). Where appropriate, *P*-values were adjusted for multiple testing using the Benjamini–Hochberg procedure to control the false discovery rate. Statistical significance differences to reference samples were indicated. Regarding statistical analysis of *in vivo* experiments, data represent the mean±S.E.M. Each group and side differences were analysed by one-way ANOVA test using the STATVIEW software (Abacus Concepts, Berkeley, CA, USA). Significance was set at *P*<0.05.
