# Supplementary material for: Neuronal hemoglobin affects dopaminergic cells' response to stress
Source: Cell Death Dis. 2017 Jan 5;8(1):e2538–. doi: 10.1038/cddis.2016.458 (PMC5386368; doi:10.1038/cddis.2016.458)
Supplement: Supplementary Information [file cddis2016458x1.pdf]

Supplementary Information

Neuronal hemoglobin affects dopaminergic cells' response to stress

Marta Codrich, Maria Bertuzzi, Roberta Russo, Margherita Francescatto, Stefano Espinoza, Lorena Zentilin, Mauro Giacca, Daniela Cesselli, Antonio Paolo Beltrami, Paolo Ascenzi, Silvia Zucchelli, Francesca Persichetti, Giampiero Leanza & Stefano Gustincich

Supplementary References

Supplementary Figure Legends

Supplementary Table

Supplementary Table S1

Supplementary Figures

Supplementary Figure S1, S2, S3, S4, S5

Supplementary References

1 Kiryk A, Sowodniok K, Kreiner G, Rodriguez-Parkitna J, Sönmez A, Górkiewicz T *et al*. Impaired rRNA synthesis triggers homeostatic responses in hippocampal neurons. *Front Cell Neurosci* 2013; **7**: 207.

2 Oie S, Matsuzaki K, Yokoyama W, Tokunaga S, Waku T, Han S-I *et al*. Hepatic rRNA transcription regulates high-fat-diet-induced obesity. *Cell Rep* 2014; **7**: 807–820.

3 Mauro C, Leow SC, Anso E, Rocha S, Thotakura AK, Tomatore L *et al*. NF-κB controls energy homeostasis and metabolic adaptation by upregulating mitochondrial respiration. *Nat Cell Biol* 2011; **13**: 1272–9.

Supplementary Figure Legends

Supplementary Figure S1. Densitometric analysis. Differentiated Hb cells and control cells were treated with MPP^{+} (a, c, e, g) or rotenone (b, d, f, h, l, m) at the indicated concentrations for 16 hours. **(a, b)** Densitometric analysis of pro-Caspase-3 expression. Pro-Caspase-3 levels were normalized to β-actin. Untreated cells was used as reference and set to 100%. (n=5, n=4) **(c, d)** Densitometric analysis of cleaved Caspase-3 expression. Cleaved Caspase-3 levels were normalized to β-actin. Untreated cells was used as reference and set to 100%. (n=5, n=6) **(e, f)** Densitometric analysis of nuclear FLAG (α-globin) expression. Nuclear α-globin levels were normalized to β-actin. Untreated cells was used as reference and set to 100%. (n=3, n=3) **(g, h)** Densitometric analysis of nuclear MYC (β-globin) expression. Nuclear β-globin levels were normalized to β-actin. Untreated cells was used as reference and set to 100%. (n=3, n=3) **(l)** Densitometric analysis of P-4EB-P1 expression. P-4EB-P1 levels were normalized to β-actin. Untreated control cells was used as reference and set to 100%. (n=3) **(m)** Densitometric analysis of LC3 II expression. LC3 II levels were normalized to β-actin. Untreated control cells was used as reference and set to 100%. (n=6) Values are mean ± SD. Data were evaluated statistically by Student's t-test. When appropriate the p-values were adjusted for multiple testing using the Benjamini-Hochberg method to control the false discovery rate. Resulting p-values are indicated.

Supplementary Figure S2. Hb toxicity doesn't depend on O_2 binding. (a, b) Characterization of Hb cells (Hb), mutated Hb cells (mut Hb) and control cells (control). **(a)** Western blot analysis of cell lysates was carried out with anti-FLAG (α-globin), anti-MYC (β-globin), anti-Hemoglobin and anti-GFP antibodies. β-actin was used as loading control. **(b)** Double immunofluorescence was performed with anti-FLAG (α-globin) and anti-MYC (β-globin) antibodies. Scale bar 10 µm. **(c)** Differentiated Hb cells, mut Hb cells and control cells were treated with rotenone at the indicated concentrations for 16 hours. Western blot analysis of cleaved Caspase-3 expression. β-actin was used as loading control. (n=4) **(d)** Differentiated mut Hb cells were treated with rotenone at the

indicated concentrations for 16 hours. Western blot analysis of cellular fractions was carried out with anti-FLAG (α-globin) antibody. Anti-TH and anti-UBF antibodies were used to visualize specifically cytoplasm and nucleus compartment respectively. β-actin was used as loading control. (n=3)

Supplementary Figure S3. Dimer structure of overexpressed Hb (αβ) is not altered in cellular model of PD. Differentiated Hb cells were treated for 16 hours with MPP^+ (a) or with rotenone (b) at the indicated concentration. **(a, b)** Lysates of Hb cells were immunoprecipitated (IP) with anti-FLAG agarose beads and bound proteins were revealed by immunoblot (IB) with anti-MYC antibody. Lysates were tested for the expression of FLAG (α-globin) and MYC (β-globin) proteins. β-actin was used as loading control. (n=4, n=4) **(c)** qRT-PCR of p53 levels. p53 levels were normalized to β-actin. Untreated control cells was used as reference and set to 1. (n=5) Values are mean ± SD. Data were evaluated statistically by Student's t-test. The p-values were adjusted for multiple testing using the Benjamini-Hochberg method to control the false discovery rate. Resulting p-values are indicated.

Supplementary Figure S4. AAV9-mediated delivery of Hb in SNpc of mouse brain. (a) PCR analysis of dissected controlateral (CL) and ipsilateral (IL) side of substantia nigra (SN) and striatum. Cortex (CTX) was used as negative control. PCR was carried out with FLAG (α-globin), MYC (β-globin) and TH primers. Additional controls were used: pcDNA3-2XFLAG-α-globin or pcDNA3.1-β-globin-MYC or DA cell sample (+) for the efficiency of PCR amplification; negative control of retrotranscription (-) was included. **(b)** Western blot analysis of dissected CL and IL side of SN and CTX. Western blot was carried out with anti-FLAG (α-globin), anti-MYC (β-globin) and anti-Hemoglobin antibodies. β-actin was used as loading control. **(c)** qRT-PCR of Hb levels. α-globin (Hba) and β-globin (Hbb) levels were normalized to β-actin. Values are expresses as fold change relative to the controlateral side of AAV9-control mice, arbitrary set to 1. (n=2) **(d)**

Immunohistochemistry of coronal sections of AAV9-Hb mouse brain. SN was stained with anti-TH antibody. Infected cells were stained with anti-FLAG (α-globin) antibody. Nuclei were visualized with DAPI. Scale bar 200 μm. Enlargement of ipsilateral side (scale bar 50 μm). **(e)** Immunohistochemistry of coronal sections of AAV9-Hb mouse brain. SN was stained with anti-TH antibody. Infected cells were stained with anti-MYC (β-globin) antibody. Nuclei were visualized with DAPI. Scale bar 200 μm. Enlargement of ipsilateral side (scale bar 50 μm). **(f)** Immunohistochemistry of coronal sections of AAV9-Hb mouse brain. SN was stained with anti-TH and anti-Hemoglobin antibodies. Scale bar 50 μm. **(g)** Double immunohistochemistry of ipsilateral side of AAV9-Hb mouse brain was performed with anti-FLAG (α-globin) and anti-MYC (β-globin) antibodies. Nuclei were visualized with DAPI. Scale bar 30 μm.

Supplementary Figure S5. Dopamine content in Hb and control cells. (a) The level of dopamine was measured in differentiated Hb and control cells by HPLC**.** (n=3) Values are mean ± SD. Data were evaluated statistically by Student's t-test. Resulting p-values are indicated.
