# Supplementary material for: Neuronal hemoglobin affects dopaminergic cells' response to stress
Source: Cell Death Dis. 2017 Jan 5;8(1):e2538–. doi: 10.1038/cddis.2016.458 (PMC5386368; doi:10.1038/cddis.2016.458)
Supplement: Supplementary Table S1 [file cddis2016458x2.pdf]

Supplementary Table S1

List of primers

Primer	Used for	Primer Sequence 5'-3'
FLAG-α-globin	PCR	fw: GCGCAAGCTTATGGACTACAAGGA<br>rev: ATATCTCGAGTTAACGGTACTTGG
β-globin-MYC	PCR	fw: GCGCATGGTGCACCTGACTGATGC<br>rev: ATATTTACAGGTCCTCCTCGCTGA
TH	PCR	fw: CCGTCTCAGAGCAGGATACC<br>rev: CGAATACCACAGCCTCCAATG
pre-rRNA pair 1[1]	qRT-PCR	fw: ACTGACACGCTGTCCTTTCC<br>rev: GACAGCTTCAGGCACCGCGA
pre-rRNA pair 2[2]	qRT-PCR	fw: CTCTTGTTCTGTGTCTGCC<br>rev: GCCCGCTGGCAGAACGAGAAG
p53[3]	qRT-PCR	fw: CACAGCGTGGTGGTACCTTA<br>rev: TCTTCTGTACGGCGGTCTCT
β-actin	qRT-PCR	fw: CACACCCGCCACCAGTTC<br>rev: CCCATTCCCACCATCACACC
α-globin H87G	Mutagenesis	fw: GCTCTGAGCGACCTGGGTGCCCACAAGCTGCGTG<br>rev: CACGCAGCTTGTGGGCACCCAGGTCGCTCAGAGC
β-globin H92G	Mutagenesis	fw: CAGCCTCAGTGAGCTCGGCTGTGACAAGCTGCATG<br>rev: CATGCAGCTTGTCACAGCCGAGCTCACTGAGGCTG
