# Supplementary figures and images for: Neuronal hemoglobin affects dopaminergic cells' response to stress
Source: Cell Death Dis. 2017 Jan 5;8(1):e2538–. doi: 10.1038/cddis.2016.458 (PMC5386368; doi:10.1038/cddis.2016.458)

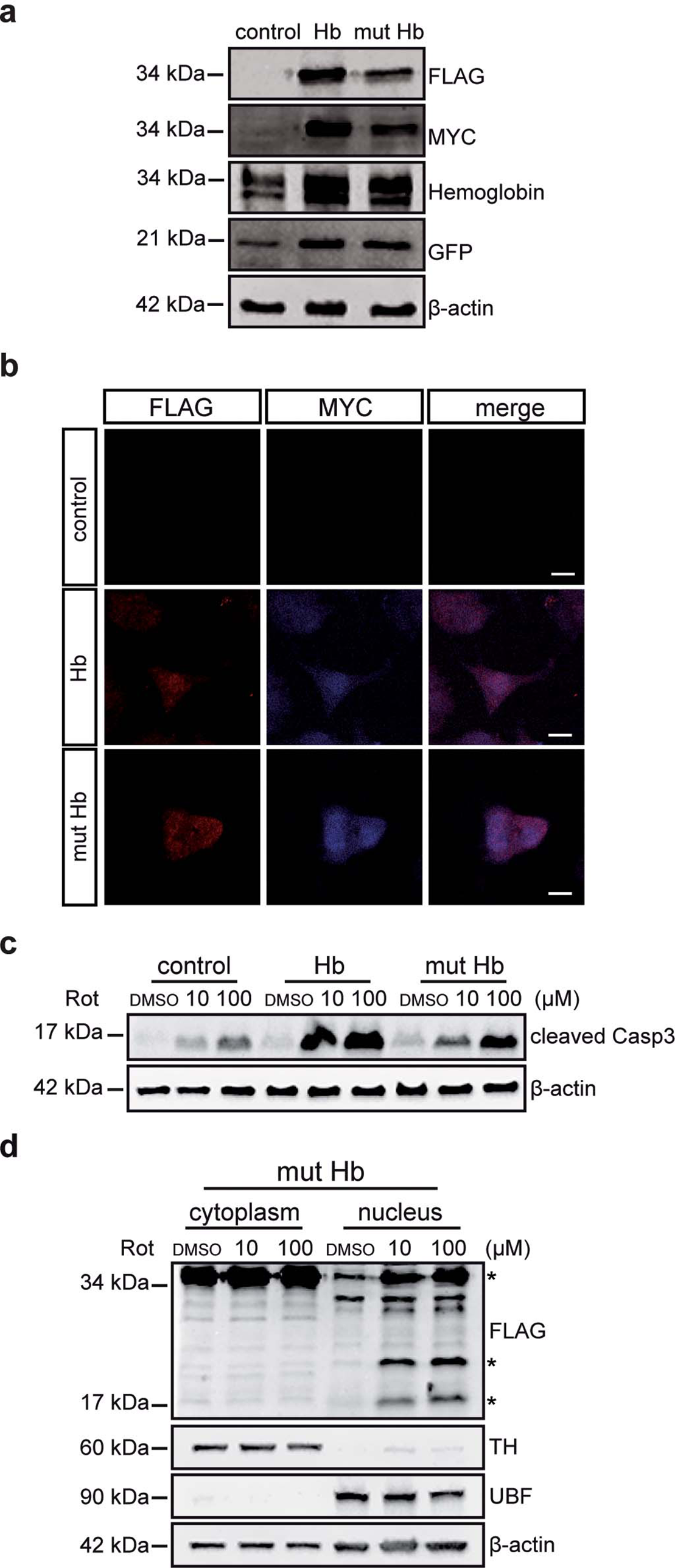

Supplement: Supplementary Figure S2 [file cddis2016458x4.tif]

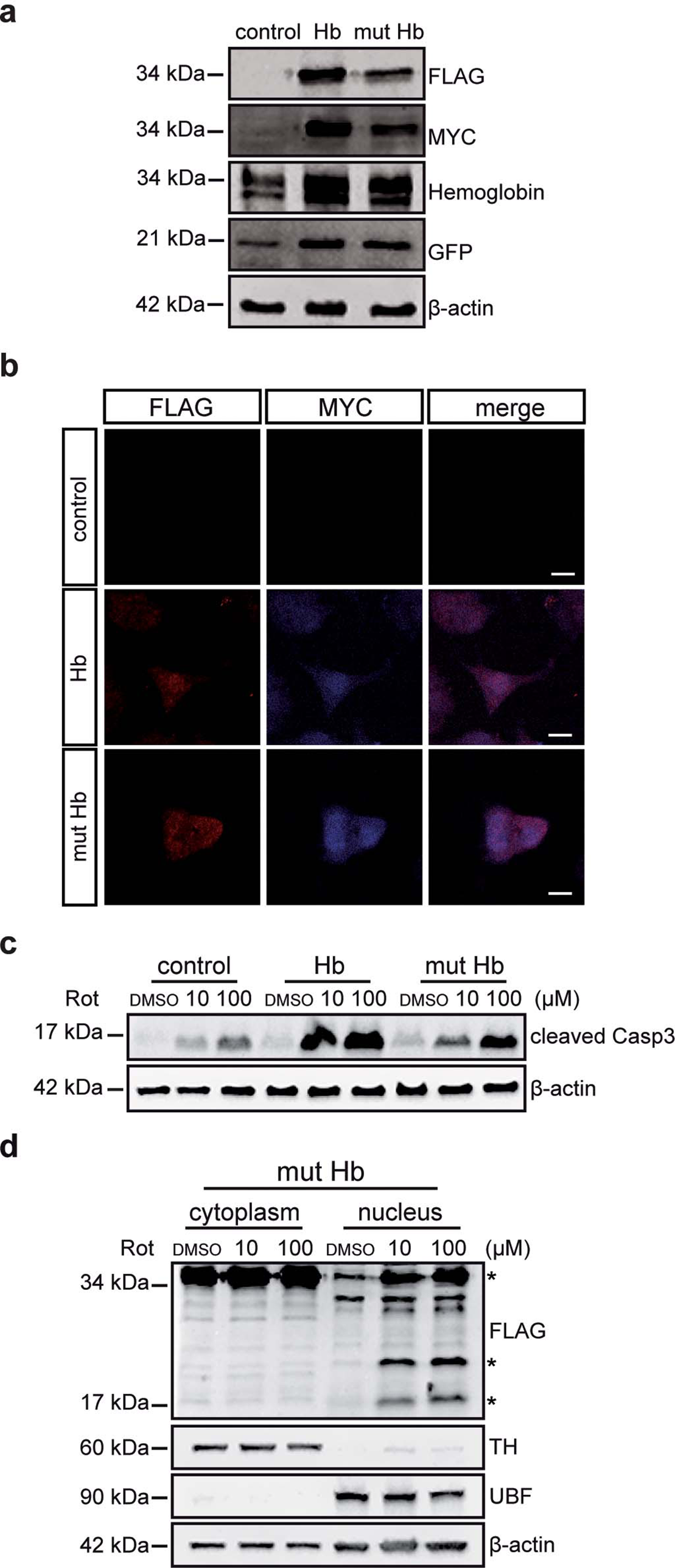

Supplement: Supplementary Figure S4 [file cddis2016458x6.tif]

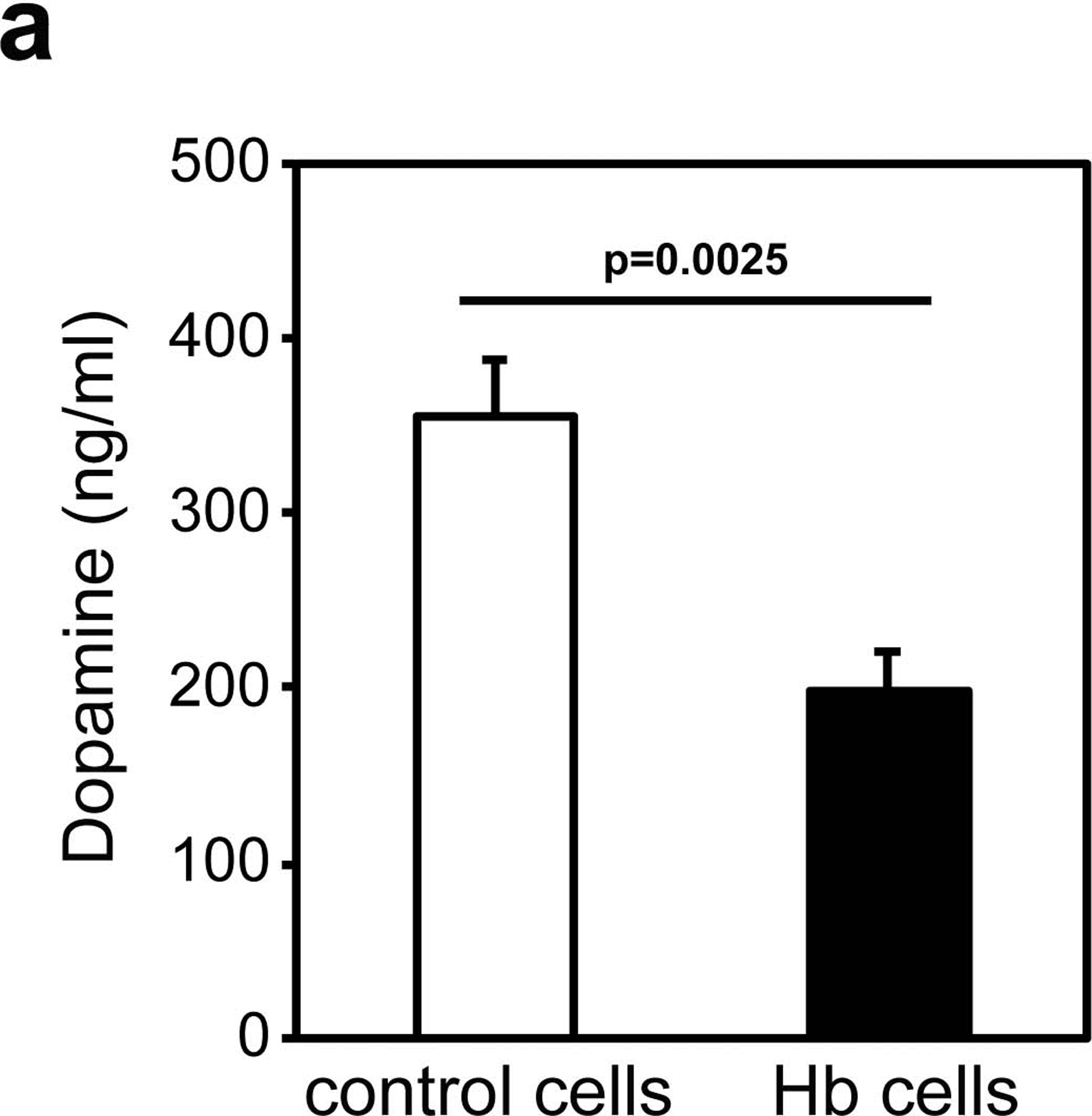

Supplement: Supplementary Figure S5 [file cddis2016458x7.tif]
